# Increased interleukin-6/C-reactive protein levels are associated with the upregulation of the adenosine pathway and serve as potential markers of therapeutic resistance to immune checkpoint inhibitor-based therapies in non-small cell lung cancer

**DOI:** 10.1136/jitc-2023-007310

**Published:** 2023-10-18

**Authors:** Abdul Rafeh Naqash, Justin D McCallen, Emma Mi, Sanna Iivanainen, Mona A Marie, Daria Gramenitskaya, James Clark, Jussi Pekka Koivunen, Shravanti Macherla, Sweta Jonnalagadda, Shanker Polsani, Rahim Ali Jiwani, Maida Hafiz, Mahvish Muzaffar, Leonardo Brunetti, Chipman R G Stroud, Paul R Walker, Kun Wang, Youngmin Chung, Eytan Ruppin, Se-Hoon Lee, Li V Yang, David J Pinato, Joo Sang Lee, Alessio Cortellini

**Affiliations:** 1 Stephenson Cancer Center, The University of Oklahoma Health Sciences Center, Oklahoma City, Oklahoma, USA; 2 Hematology / Oncology Division, East Carolina University, Greenville, South Carolina, USA; 3 Department of Internal Medicine, University of North Carolina at Chapel Hill, Chapel Hill, North Carolina, USA; 4 Brody School of Medicine, East Carolina University, Greenville, NC, USA; 5 Department of Surgery and Cancer, Hammersmith Hospital Campus, Imperial College London, London, UK; 6 Oncology and Radiation Department, Oulu University Hospital, University of Oulu, MRC Oulu, Oulu, Finland; 7 Department of Internal Medicine, East Carolina University, Greenville, NC, USA; 8 Division of Pulmonary Critical Care, University of Oklahoma Health Sciences Center, Oklahoma City, Oklahoma, USA; 9 Division of Pulmonary and Critical Care, East Carolina University, Greenville, NC, USA; 10 Operative Research Unit of Medical Oncology, Fondazione Policlinico Universitario Campus Bio-Medico, Via Alvaro del Portillo 200, Roma, Italy, Italy; 11 Genentech, South San Francisco, California, USA; 12 Circulogene, Birmingham, Alabama, USA; 13 Cancer Data Science Lab, National Cancer Institute, National Institute of Health, Bethesda, Maryland, USA; 14 Department of Artificial Intelligence, Sungkyunkwan University, Suwon, Reuplic of Korea; 15 Division of Hematology-Oncology, Department of Medicine, Samsung Medical Center School of Medicine, Sungkyunkwan University, Seoul, Republic of Korea; 16 Department of Health Sciences and Technology, Samsung Advanced Institute of Health Sciences and Technology, Sungkyunkwan University, Seoul, Republic of Korea; 17 Division of Oncology, Department of Translational Medicine, University of Piemonte Orientale, Novara, Italy; 18 Department of Precision Medicine, School of Medicine, Sungkyunkwan University, Suwon, Republic of Korea; 19 Department of Digital Health, Samsung Advanced Institute of Health Sciences and Technology, Sungkyunkwan University, Seoul, Republic of Korea

**Keywords:** Adenosine, Tumor Microenvironment, Immune Checkpoint Inhibitors, Lung Neoplasms, Inflammation

## Abstract

**Background:**

Systemic immune activation, hallmarked by C-reactive protein (CRP) and interleukin-6 (IL-6), can modulate antitumor immune responses. In this study, we evaluated the role of IL-6 and CRP in the stratification of patients with non-small cell lung cancer (NSCLC) treated with immune checkpoint inhibitors (ICIs). We also interrogated the underlying immunosuppressive mechanisms driven by the IL-6/CRP axis.

**Methods:**

In cohort A (n=308), we estimated the association of baseline CRP with objective response rate (ORR), progression-free survival (PFS), and overall survival (OS) in patients with NSCLC treated with ICIs alone or with chemo-immunotherapy (Chemo-ICI). Baseline tumor bulk RNA sequencing (RNA-seq) of lung adenocarcinomas (LUADs) treated with pembrolizumab (cohort B, n=59) was used to evaluate differential expression of purine metabolism, as well as correlate *IL-6* expression with PFS. CODEFACS approach was applied to deconvolve cohort B to characterize the tumor microenvironment by reconstructing the cell-type-specific transcriptome from bulk expression. Using the LUAD cohort from The Cancer Genome Atlas (TCGA) we explored the correlation between *IL-6* expression and adenosine gene signatures. In a third cohort (cohort C, n=18), plasma concentrations of CRP, adenosine 2a receptor (A2aR), and IL-6 were measured using ELISA.

**Results:**

In cohort A, 67.2% of patients had a baseline CRP≥10 mg/L (CRP-H). Patients with CRP-H achieved shorter OS (8.6 vs 14.8 months; p=0.006), shorter PFS (3.3 vs 6.6 months; p=0.013), and lower ORR (24.7% vs 46.3%; p=0.015). After adjusting for relevant clinical variables, CRP-H was confirmed as an independent predictor of increased risk of death (HR 1.51, 95% CI: 1.09 to 2.11) and lower probability of achieving disease response (OR 0.34, 95% CI: 0.13 to 0.89). In cohort B, RNA-seq analysis demonstrated higher *IL-6* expression on tumor cells of non-responders, along with a shorter PFS (p<0.05) and enrichment of the purinergic pathway. Within the TCGA LUAD cohort, tumor *IL-6* expression strongly correlated with the adenosine signature (R=0.65; p<2.2e−16). Plasma analysis in cohort C demonstrated that CRP-H patients had a greater median baseline level of A2aR (6.0 ng/mL vs 1.3 ng/mL; p=0.01).

**Conclusions:**

This study demonstrates CRP as a readily available blood-based prognostic biomarker in ICI-treated NSCLC. Additionally, we elucidate a potential link of the CRP/IL-6 axis with the immunosuppressive adenosine signature pathway that could drive inferior outcomes to ICIs in NSCLC and also offer novel therapeutic avenues.

**WHAT IS ALREADY KNOWN ON THIS TOPIC**

- C-reactive protein (CRP) is a standard clinical marker of inflammation that can be synthesized in response to interleukin (IL)-6, which has been widely associated with poor outcomes in non-small cell lung cancer (NSCLC).

**WHAT THIS STUDY ADDS**

- The study confirms baseline CRP and IL-6 as prognostic blood-based biomarkers in patients with NSCLC treated with immune checkpoint inhibitors (ICIs)-based therapies and indicates an association between peripheral blood CRP, IL-6, and adenosine 2a receptor (A2aR) levels, tumorous IL-6 expression, and an immunosuppressive adenosine signature.

**HOW THIS STUDY MIGHT AFFECT RESEARCH, PRACTICE OR POLICY**

- Our data suggests a potential role for A2aR inhibition in combination with IL-6 receptor blockade in ICI-treated patients with a CRP-high phenotype.

## Background

The benefit of immune checkpoint inhibitors (ICIs) as monotherapy or combination therapy has rapidly reshaped the treatment landscape of non-small cell lung cancer (NSCLC). Currently, ICIs alone or combined with chemotherapy (Chemo-ICI) have received US Food and Drug Administration approvals across many settings in NSCLC, ranging from neoadjuvant to metastatic.^1–5^ However, while programmed death-ligand 1 (PD-L1) expression and tumor mutational burden have shown promise as biomarkers in select settings, due to inherent challenges associated with their quantification, they have failed to consistently and conclusively discriminate treatment benefits to ICIs alone or with Chemo-ICI.^6 7^ Meanwhile, we and others have previously used the combination of routinely available pro-inflammatory biomarkers, including peripheral blood neutrophilia, lymphopenia, and high lactate dehydrogenase, to develop inflammation-based prognostic models to aid in patient stratification and clinical benefit from immunotherapy-based treatments.^8–14^

Inflammation is essential in cancer pathogenesis and fosters an oncogenic microenvironment favorable for tumor survival and proliferation.^15^ C-reactive protein (CRP) is a standard clinical marker of inflammation. CRP is the principal member of the pentraxin family, a pattern-recognition receptor protein. It is the prototypical acute phase reactant produced in response to pro-inflammatory stimuli of various origins, including cancer.^16^ Although primarily produced in the liver, CRP can also be synthesized in macrophages, endothelial cells, and lymphocytes in response to interleukin (IL)6, tumor necrosis factor, and IL-1β via transcriptional activation of the signal transducer and activator of transcription factor 3 (STAT3).^17^ In NSCLC, high-serum CRP has been widely associated with poor outcomes.^18–21^ Prior retrospective data sets from our group and others have shown that elevated levels of baseline CRP are associated with worse outcomes in NSCLC and other tumors treated with single-agent ICI in the second-line setting.^10 22–25^ However, most of these studies are limited due to the small sample size, single institutional data, or absence of patients treated with front-line chemo-immunotherapy combinations.

IL-6 is essential in mediating inflammation and inducing CRP synthesis and release from the liver. While secreted in response to a plethora of pro-inflammatory stimuli, serum CRP concentration is tightly correlated with circulating IL-6 levels. Patients with melanoma with higher circulating CRP and IL-6 levels were reported to have lower response rates and shorter survival to ICI therapy, highlighting a putative role for the IL-6/CRP axis in determining resistance to immunotherapy.^26 27^ Among the proposed mechanism underlying this association, IL-6-mediated induction of ectonucleotidases such as CD39 and CD73 can indirectly augment the production of extracellular adenosine, a known immunosuppressive metabolite acting via the adenosine 2a receptor (A2aR), and thus attenuate antitumor immune responses.^28–30^ CRP can upregulate hypoxia-inducible factors, which regulate immunosuppressive adenosine production via CD39 and CD73 and A2aR expression on cancer and immune cells independent of IL-6.^31–34^

Based on these recent observations, we hypothesized that elevated baseline IL-6 in blood and tumor tissue is an adverse prognostic marker in NSCLC treated with ICIs and has therapeutic implications. Furthermore, we posited that one possible mechanism of IL-6-mediated immunotherapy resistance could be through the adenosine pathway and A2aR-mediated immunosuppression.

This multicenter international observational study primarily explored the differences in clinical outcomes to ICI alone or Chemo-ICI in patients with advanced NSCLC via baseline peripheral blood CRP level at ICI initiation. Secondarily, in a distinct cohort of patients with advanced NSCLC, we used transcriptomic analysis to demonstrate the correlation of tumorous *IL-6* expression with NSCLC treatment response and survival with single-agent ICI. Furthermore, in another pilot cohort of ICI-treated NSCLC, we correlated serum plasma levels for IL-6 with CRP and A2aR in the blood. Lastly, we correlated a composite adenosine signature with tumorous IL-6 expression in NSCLC to better understand the potential role of the IL-6-adenosine axis in mediating poor outcomes to ICIs.

## Methods

### Study criteria of cohort #1

Patients with NSCLC treated with either ICIs alone or Chemo-ICI between 2015 and 2019 at four (one US and three European) academic centers were identified. Patients were included if they were ≥18 years old, had pathologically confirmed advanced NSCLC, and received ≥1 dose of ICI-based therapies with a follow-up cut-off of April 2020. Patients with locally advanced disease (stage IIIb according to the seventh edition of the American Joint Committee on Cancer (AJCC) staging system, for those treated before 2018 and stage IIIb/IIIc according to the eighth edition of the AJCC for those treated from 2018 onwards), not amenable to radical treatment including surgery and definitive chemo-radiation receiving ICI-based therapy were considered eligible, while patients treated with maintenance durvalumab after chemo-radiation were excluded.

CRP collected from peripheral blood up to 2 weeks before starting ICI-based treatments was considered as baseline. A CRP cut-off of 10 mg/L was used to define two groups, that is, CRP-high = ≥10 mg/L and CRP-low = <10 mg/L. This cut-off has been previously described as a surrogate for systemic inflammation in more extensive population-based studies and poor prognosis in patients with cancer and has been validated in the context of the modified Glasgow Prognostic Score in patients treated with ICI-based regimens.^35–37^ In addition, the 10 mg/L cut-off has been used in several studies assessing patients with NSCLC treated with both chemotherapy and ICIs.^38–40^ CRP was assessed in clinical practice, using immunoturbidimetry (three centers) or antibody-based nephelometric assays (one center).

This study aimed to explore the impact of baseline CRP levels on the clinical outcomes of patients with advanced NSCLC receiving ICI-based therapies. Clinical endpoints of interest included overall survival (OS), progression-free survival (PFS), and objective response rate (ORR). Patients were evaluated for disease response during treatment every 12 weeks (±7 days); investigators were asked to provide disease assessments following Response Evaluation Criteria in Solid Tumors (RECIST) criteria V.1.1. PFS and OS were measured from treatment initiation to disease progression or death, respectively. Patients without documented disease progression were censored on the date of the last imaging follow-up (April 1, 2020).

We first estimated the associations between baseline CRP levels (CRP-high and CRP-low) and patients’ characteristics. We then evaluated the role of CRP within the whole study population with univariable and multivariable analyses using a fixed multivariable regression model. As an additional endpoint, we explored the potential differential impact of CRP on patients treated with either ICI alone or Chemo-ICI combination.

Covariates were chosen with a clinical prioritization, considering their already proven prognostic role in patients with NSCLC receiving ICIs, and selected by their availability. Included covariates were: PD-L1 expression on tumor cells (0 vs 1–49% vs ≥50% vs unknown), smoking status (current/former smokers vs never smokers vs unknown), Eastern Cooperative Oncology Group performance status (ECOG-PS) (0–1 vs ≥2), cancer stage (IIIb/IIIc vs IV), liver metastases (yes vs no), brain metastases (yes vs no), bone metastases (yes vs no), sex (male vs female), age (≥65 vs <65 years old), primary tumor histology (squamous vs non-squamous), and treatment line (first vs non-first).

### Statistical analysis

Descriptive statistics were used to report baseline clinic-pathologic characteristics. X^2^/Fisher’s exact test (as appropriate) was used to determine associations between CRP/therapeutic modality and the categorical covariates of interest and for the univariable analysis of ORR.

The median period of follow-up was computed with the reverse Kaplan-Meier method. Univariable analyses of PFS and OS were performed using the Kaplan-Meier method and the log-rank test. A pooled fixed multivariable model including the key covariates of interest and CRP categories was used to estimate the potentially independent role of CRP. Cox proportional hazards regression was used for the multivariable analysis of PFS (risk of disease progression/death) and OS (risk of death) and to compute all the HRs with CIs, while the logistic regression was used for the multivariable analysis of ORR (probability of achieving a disease response) presented through ORs with 95% CI. Considering that the data source consisted of four different centers where different methodologies for CRP assessment were used in clinical practice a conditional interpretation for participating center by using frailty models was applied to correct all the 95% CI of multivariable Cox regressions, while a clustered robust correction for participating center by using the *sandwich* and *lmtest* packages was applied to all the 95% CI of logistic regression. Variables with ≥5% missingness were included in the “unknown” category and those with data missingness <5% were included among the reference categories.

Analyses were performed using the RStudio software, R Core Team 2021 (R: A language and environment for statistical computing. R Foundation for Statistical Computing, Vienna, Austria), and the MedCalc Statistical Software V.20 (MedCalc Software, Ostend, Belgium; https://www.medcalc.org; 2021).

### Study criteria of cohort #2 (Korean cohort)

An unpublished cohort of patients with advanced NSCLC treated with pembrolizumab alone was identified (n=59). The median age of patients was 61.4 years. Patients were included if they were ≥18, had pathologically confirmed advanced NSCLC, and received ≥1 dose of pembrolizumab. All the patients in this cohort had lung adenocarcinoma. Prior to treatment initiation, tumor biopsy samples were obtained. RNA was purified from formalin-fixed paraffin-embedded or fresh tumor samples using the AllPrep DNA/RNA Mini Kit (QIAGEN, USA). The RNA concentration and purity were measured using the NanoDrop and Bioanalyzer (Agilent, USA). The library was prepared following the manufacturer’s instructions using the RNA Access Library Prep Kit (Illumina, USA).

### Deconvolution of bulk RNA-seq

Previously, we used CODEFACS designed to characterize the tumor microenvironment (TME) by reconstructing the cell-type-specific transcriptome from bulk expression.^41^ As input, CODEFACS takes bulk RNA sequencing (RNA-seq) expression values of tumor samples and cell-type-specific molecular signature profiles to generate cell-type-specific transcriptomic levels with confidence estimates. The CODEFACS approach was applied to deconvolve the The Cancer Genome Atlas (TCGA) samples and the ICI-treated lung cancer samples (South Korean cohort).

### Patient survival and correlation analysis

Tumor response was assessed by physicians using the RECIST V.1.1 criteria. Complete or partial responses of tumors were counted as responders. Stable disease or progression was measured as non-responders. We performed Kaplan-Meier analysis to evaluate the association of IL-6 gene expression on tumor cells as determined by CODEFACS with PFS. We compared the survival of patients treated with single-agent pembrolizumab having high IL-6 expression on the tumor cells (n=24; greater than the median) versus low IL-6 in cancer cells (n=35; lower than the median) using the log-rank test. In addition, we performed pathway enrichment analysis using Gorrilla^42^ for the genes that were differentially expressed in the cancer-specific IL-6 high versus IL-6 low samples in the same cohort. This pathway enrichment analysis identifies those differentially active pathways in cancer-specific IL-6 high samples versus IL-6 low samples. The correlation between the adenosine pathway and IL-6 expression was quantified using TCGA bulk RNA-seq data of lung adenocarcinoma, where the adenosine pathway signature was obtained from previously published data.^43^

### Study criteria for cohort #3

In a separate prospective pilot project at East Carolina University, we enrolled 18 patients with advanced NSCLC between December 2016 and April 2019 treated with single-agent ICI or Chemo-ICI. We isolated plasma samples from patients before ICI initiation and after each treatment. Baseline plasma concentrations of A2aR and IL-6 were measured using ELISA. The baseline level of CRP was obtained from pretreatment peripheral blood on C1D1 (± 3 days) of ICI initiation. We assessed the relationship between CRP, IL-6, and A2aR plasma levels. Statistical analyses were performed using the Mann-Whitney test and linear regression analysis.

### Blood collection, processing, and ELISA quantification

In the separate cohort of 18 patients from East Carolina University who were enrolled in a prospective biomarker-based study, whole blood samples were collected in EDTA-treated tubes, and blood plasma was isolated using the Ficoll/Hypaque density gradient centrifugation method. For detecting circulating IL-6 and soluble A2aR in the plasma, human IL-6 Quantikine ELISA Kit (R&D Systems, Minneapolis, Minnesota, USA; D6050), human adenosine A2A Receptor ELISA kit, (LSBio, Seattle, Washington, USA; LS-F10701), were used according to manufacturer’s protocol. The absorbance at 450 nm was measured using Molecular Devices SpectraMax M2. The concentration of IL-6 and A2aR were determined using a four-parameter regression curve of the absorbance compared with the standards. ELISAs were performed in duplicate for each sample.

## Results

### Cohort #1: CRP-H is a poor prognostic factor in advanced NSCLC treated with ICIs

Overall, 420 patients with NSCLC were screened. After the exclusion of 103 patients for whom baseline CRP was not available, and 8 patients with stage III NSCLC treated with consolidation durvalumab after definitive chemo-radiation therapy, 308 patients were included in the final analysis as outlined in online supplemental figure 1. Baseline clinic-pathologic features of the 308 patients who were included are summarized in table 1 and online supplemental table 1. The median CRP was 21.0 mg/L. One-third of patients (n=101) had CRP ≤10 mg/L (CRP-low). At ICI initiation, most patients had stage IV NSCLC (77.9%). In the entire cohort, administered ICIs were nivolumab (184, 59.7), pembrolizumab (52, 16.9%), atezolizumab (22, 7.1%), nivolumab–ipilimumab combination (2, 0.6%) and Chemo-ICI combinations (48, 15.6%). CRP-high did not show an association with age ≥65, gender, histology, smoking status, ECOG-PS, tumor stage, liver metastases, brain metastases, liver metastases, PD-L1 expression, treatment line and treatment regimen (table 1).

10.1136/jitc-2023-007310.supp2 Supplementary data

**Table 1:** Baseline patient characteristics of cohort #1 for the overall population and according to CRP category

Variable	Overall 308 N (%)	Low 101 N (%)	High 207 N (%)	P value
CRP (mg/dL)				
Median (range)	21.2 (0.1–302.4)	3.8 (0.1–10)	36.0 (10.1–302.4)	–
Age				
Median (range) ≥65 years old <65 years old	65 (35–87) 140 (45.5) 168 (54.5)	65 (37–87) 49 (48.5) 52 (51.5)	66 (35–86) 91 (44.0) 116 (56.0)	0.4519
Gender				
Female Male	111 (36.0) 197 (64.0)	43 (42.6) 58 (57.4)	68 (32.9) 139 (67.1)	0.0957
Histology				
Non-squamous Squamous	196 (63.6) 112 (36.4)	72 (71.3) 29 (28.7)	124 (59.9) 83 (40.1)	0.0516
Smoking at ICI Initiation				
Current/former smokers Never smokers Unknown	190 (61.7) 13 (4.2) 105 (34.1)	61 (60.4) 6 (5.9) 34 (33.7)	129 (62.3) 7 (3.4) 71 (34.3)	0.5764
ECOG-PS				
0 1 ≥2 Unknown	67 (21.8) 162 (52.6) 72 (23.4) 7 (2.3)	29 (28.7) 44 (43.6) 25 (24.8) 3 (3.0)	38 (18.4) 118 (57.0) 47 (22.7) 4 (1.9)	0.1059
Cancer stage				
IIIb/IIIc IV	68 (22.1) 240 (77.9)	27 (26.7) 74 (73.3)	41 (19.8) 166 (80.2)	0.1696
Liver metastasis at ICI initiation				
No Yes	269 (87.3) 39 (12.7)	89 (88.1) 12 (11.9)	180 (87.0) 27 (13.0)	0.7737
Brain metastasis at ICI initiation				
No Yes	243 (78.9) 65 (21.1)	75 (74.3) 26 (25.7)	169 (81.2) 39 (18.8)	0.1641
Bone metastasis at ICI initiation				
No Yes	222 (72.1) 86 (27.9)	77 (76.2) 24 (23.8)	145 (70.0) 62 (30.0)	0.2564
PD-L1%				
0 1–49 ≥50 Unknown	47 (15.3) 45 (14.6) 77 (25.0) 139 (45.1)	16 (15.8) 17 (16.8) 27 (26.7) 41 (40.6)	31 (15.0) 28 (13.5) 50 (24.2) 98 (47.3)	0.7040
Treatment line				
First Non-first	77 (25.0) 231 (75.0)	26 (25.7) 75 (74.3)	51 (24.6) 156 (75.4)	0.8338
Treatment regimen				
Chemo-ICI ICI alone	48 (15.6) 260 (84.4)	18 (17.8) 83 (82.2)	30 (14.5) 177 (85.5)	0.4503
Treatment regimen				
Atezolizumab Nivolumab–ipilimumab Nivolumab Pembrolizumab Chemo-ICI	22 (7.1) 2 (0.6) 184 (59.7) 52 (16.9) 48 (15.6)	–	–	–

The number in brackets next to each variable is the valid total for each category.

Chemo-ICI, chemotherapy and immune checkpoint inhibitor; CRP, C-reactive protein; ECOG-PS, Eastern Cooperative Oncology Group Performance Status; ICI, immune checkpoint Inhibitor; PD-L1, programmed death-ligand 1.

**Figure 1:**
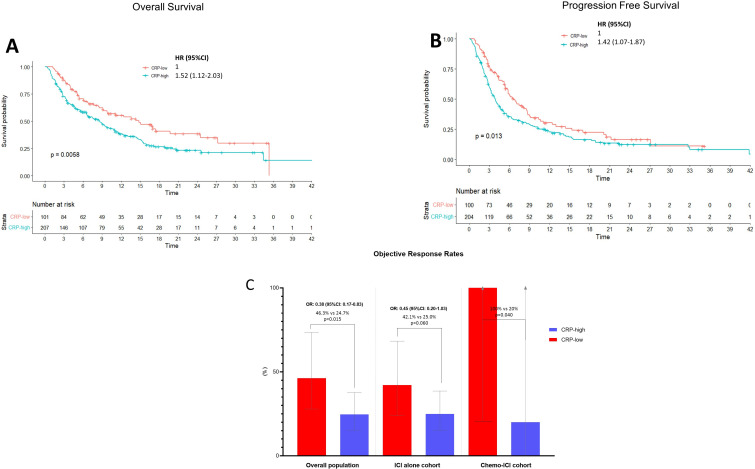
Kaplan-Meier survival estimates according to the CRP category among the whole study population. (A) Overall survival (OS). The median OS of patients with CRP-high was 8.6 months (95% CI: 6.6 to 11.0; 137 events), while the median OS of patients with CRP-low was 14.8 months (95% CI: 9.8 to 27.2; 52 events) (p=0.006) HR=1.52 (95% CI: 1.12 to 2.03). (B) Progression-free survival (PFS). The median PFS of patients with CRP-high was 3.3 months (95% CI: 3.3 to 4.7; 168 events) while the median PFS of CRP-low patients was 6.6 months (95% CI: 5.3 to 8.7; 71 events) (p=0.013) HR=1.42 (95% CI: 1.07 to 1.87). (C) Objective response rate (ORR) analysis according to the CRP category among the whole study population and the ICI alone, Chemo-ICI cohorts separately. For the overall population, among the evaluable CRP-low patients, the ORR was 46.3% (95% CI: 27.9 to 73.5), while among CRP-high was 24.7% (95% CI: 15.3 to 37.7); p=0.015. Among the ICI alone cohort, patients with CRP-low experienced higher ORR in comparison to patients with CRP-high (42.1% vs 25.0%, p=0.060). Similarly, among the Chemo-ICI cohort patients with CRP-low achieved an ORR compared with patients with CRP-high (100% vs 20%, p=0.040). CRP, C-reactive protein; Chemo-ICI: chemotherapy and immune checkpoint inhibitor.

The median follow-up for the whole study population was 17.5 months (95% CI: 16.6 to 20.0). The median OS of patients with CRP-high was 8.6 months (95% CI: 6.6 to 11.0; 137 events), while the median OS of patients with CRP-low was 14.8 months (95% CI: 9.8 to 27.2; 52 events; p=0.006; HR=1.52 (95% CI: 1.12 to 2.03) (figure 1A). In addition, the median PFS of patients with CRP-high was 3.3 months (95% CI: 3.3 to 4.7; 168 events), while the median PFS of CRP-low patients was 6.6 months (95% CI: 5.3 to 8.7; 72 events); p=0.013; (HR 1.42; 95% CI 1.07 to 1.87) (figure 1B).

Among the evaluable CRP-low patients, the ORR was 46.3% (95% CI: 27.9 to 73.5), while among CRP-high was 24.7% (95% CI: 15.3 to 37.7); p=0.015 (figure 1C).

We performed a pooled fixed multivariable analysis for ORR, PFS, and OS, reported in table 2, which confirmed the significant detrimental effect of baseline CRP-high on the probability of achieving a disease response (OR 0.34, 95% CI: 0.13 to 0.89) and the risk of death (HR 1.51, 95% CI: 1.09 to 2.11). A similar trend was observed in the risk of disease progression/death (HR 1.31, 95% CI: 0.99 to 1.75).

**Table 2:** Pooled multivariable analyses for objective response rate, progression-free survival and overall survival

Variable	Multivariable analysis		
	Objective response rate	Progression-free survival	Overall survival
	OR (95% CI)	HR (95% CI)	HR (95% CI)
CRP at ICI Initiation			
High vs low	0.34 (0.13 to 0.89)	1.31 (0.99 to 1.75)	1.51 (1.09 to 2.11)
Histology			
Squamous vs non-squamous	1.32 (0.94 to 1.83)	1.18 (0.87 to 1.59)	1.32 (0.94 to 1.83)
Sex			
Male vs female	1.79 (0.62 to 5.17)	1.10 (0.82 to 1.46)	0.96 (0.70 to 1.31)
Age			
≥65 vs <65 years old	0.97 (0.37 to 2.58)	1.08 (0.82 to 1.42)	1.23 (0.91 to 1.67)
Treatment line			
Non-first vs first-line	1.31 (0.39 to 4.45)	1.60 (0.96 to 2.66)	1.75 (0.90 to 3.37)
PD-L1%			
0 1–49 ≥50 Unknown	1 2.83 (0.24 to 32.86) 22.26 (1.91 to 259.19) 8.97 (0.81 to 99.16)	1 0.67 (0.39 to 1.14) 0.65 (0.38 to 1.09) 0.81 (0.53 to 1.26)	1 0.85 (0.46 to 1.55) 0.85 (0.46 to 1.57) 1.05 (0.65 to 1.71)
Smoking at ICI initiation			
Current/former smokers Never smokers Unknown	1 3.14 (0.29 to 33.01) 1.04 (0.26 to 4.07)	1 1.13 (0.52 to 2.45) 1.01 (0.73 to 1.39)	1 0.99 (0.38 to 2.57) 0.91 (0.63 to 1.30)
ECOG-PS			
0–1 vs ≥2	0.51 (0.09 to 2.80)	1.38 (1.01 to 1.89)	2.12 (1.51 to 2.98)
Cancer stage			
IV vs III	0.44 (0.12 to 1.63)	1.57 (1.08 to 2.28)	1.45 (0.95 to 2.20)
Treatment modality			
ICI alone vs CT-ICI	0.45 (0.07 to 3.02)	1.33 (0.73 to 2.40)	1.69 (0.76 to 3.72)
Liver metastasis at ICI initiation			
Yes vs no	1.52 (0.32 to 7.21)	1.16 (0.76 to 1.76)	1.01 (0.64 to 1.57)
Brain metastasis at ICI initiation			
Yes vs no	0.49 (0.12 to 1.98)	1.02 (0.71 to 1.48)	1.42 (0.96 to 2.11)
Bone metastasis at ICI initiation			
Yes vs no	0.34 (0.10 to 1.14)	1.13 (0.81 to 1.57)	1.23 (0.85 to 1.77)

CRP, C-reactive protein; CT, chemotherapy; ECOG-PS, Eastern Cooperative Oncology Group performance status; ICI, immune checkpoint inhibitor; PD-L1, programmed death-ligand 1.

In an exploratory analysis, we evaluated the influence of baseline CRP levels (high vs low) across the two main immunotherapy strategies in NSCLC, that is, Chemo-ICI versus ICI alone. Online supplemental figure 2 reports the Kaplan-Meier survival analyses for PFS and OS across the ICI alone and Chemo-ICI cohorts. Among the ICI alone cohort CRP-high patients experienced a shorter OS (HR 1.51, 95% CI: 1.09 to 2.11) and PFS (HR 1.42, 95% CI: 1.08 to 1.91) compared with CRP-low patients, whist a non-significant trend towards shorter OS (HR 2.38, 95% CI: 0.66 to 8.57) and PFS (HR 1.29, 95% CI: 0.59 to 2.88) was reported among the Chemo-ICI cohort. Among the ICI alone cohort, patients with CRP-low experienced higher ORR in comparison to patients with CRP-high (42.1% vs 25.0%, p=0.060). Similarly, among the Chemo-ICI cohort patients with CRP-low achieved an improved ORR compared with patients with CRP-high (100% vs 20%, p=0.040, figure 1C).

### Elevated IL-6 expression on the tumor correlates with poor outcomes to ICIs

In cohort #2 (Korean cohort) of patients with lung adenocarcinoma treated with pembrolizumab (n=59), the median age was 64.1. Approximately 64.4% (n=38) were men. A majority of patients (n=49, 84.5%) were PD-L1 positive (>1%) using the 22c3 antibody. Clinical characteristics of this cohort are shown in online supplemental table 2.

**Figure 2:**
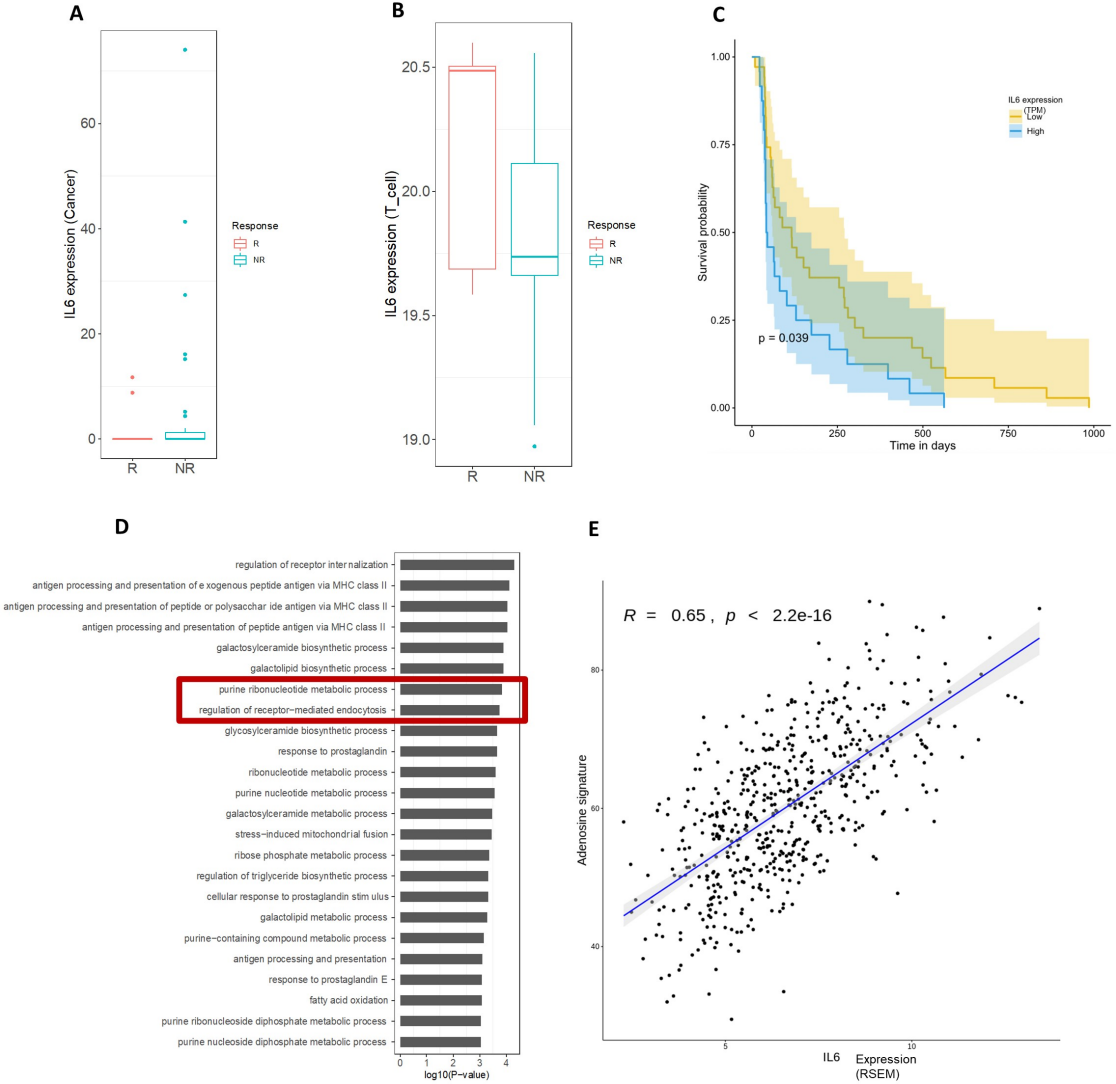
Data from cohort B comprising lung adenocarcinoma tumors. (A) Boxplot showing that the IL-6 expression in cancer cell is significantly higher in non-responders (blue; n=44) versus responders (red; n=15) with Wilcoxon rank-sum p<0.08, whereas the IL-6 expression in T-cell (B) shows the opposite trend (Wilcoxon rank-sum p<0.08). Non-responders (NR) included SD/PD and responders (R(included tumors with PR/CR. (C) Kaplan-Meier plot of the progression-free survival of patients with ICT-treated lung cancer with high IL-6 expression (transcripts per million; TPM) in cancer cells (blue; n=24) versus low IL-6 expression in cancer (yellow; n=35). The median survival difference was 81 days, p=0.039. (D) (Korean cohort) Pathway enrichment analysis of the genes upregulated in high IL-6 expression (in cancer cells) versus low IL-6 expression (in cancer cells). The pathways are listed in the vertical axis, and the enrichment p values are denoted in the horizontal axis (−log(P)). Large number on the horizontal axis denotes a more significant enrichment. (E) (TCGA analysis) X-axis shows IL-6 expression and Y-axis shows the adenosine pathway signature from a study by Araki *et al*.^39^ The Spearman rank correlation coefficient and the associated p value is noted at the top of the figure. CR, complete response; IL, interleukin; MHC, major histocompatibility complex; PD, progressive disease; PR, partial response; RSEM, RNA-seq by expectation-maximization; SD, stable disease; TCGA, The Cancer Genome Atlas.

Because IL-6 is the source key stimulus of CRP production, we hypothesized that high tumorous IL-6 could be associated with worse patient outcomes. The IL-6 expression of tumor and T cells from patients with NSCLC (Korean cohort) was determined as described above (see Study criteria of cohort #2 (Korean cohort)). Patients were stratified into two groups: above median IL-6 expression or below IL-6 median expression. Patients who did not respond to ICI (pembrolizumab) had significantly higher median IL-6 tumor expression than those who responded to treatment (figure 2A). Using the deconvolution approach from bulk-RNA seq, we observed the reverse trend when examining the IL-6 expression in T cells, wherein patients that responded to ICI treatment had high IL-6 expression in intratumoral T-cells (figure 2B). Unfortunately, the present deconvolution algorithm does not have such capability to resolve the T-cell phenotypes involved in differentiating these outcomes. Patients with tumor cells expressing high IL-6 had a significantly shorter PFS than those with tumors producing less IL-6 (n_high_=24 vs n_low_=35, median survival difference=81 days, p=0.039) (figure 2C). This data demonstrates that baseline IL-6 expression on the tumor could be an essential mediator in determining ICI outcomes in NSCLC.

We next performed pathway enrichment analysis to identify other potential drug targets and actionable pathways associated with increased IL-6 production (Korean cohort). Compared with tumors with low IL-6 expression (below median), those with high IL-6 expression (above median) had a differentially increased expression of several metabolic pathways, including the purine metabolism pathway that is critical to adenosine receptor signaling and physiology^44^ (figure 2D). Furthermore, incorporating the adenosine pathway signature recently described by Fong *et al*, we observed a strong correlation between IL-6 expression in cancer cells and its associated adenosine signature using bulk-RNA-seq data for NSCLC from TCGA with a coefficient correlation of 0.65 (figure 2E). These analyses reveal an additional immunosuppressive pathway related to the CRP and IL-6 axis in NSCLC.

### Patients with NSCLC with CRP-high have elevated plasma IL-6 and A2aR levels

**Figure 3:**
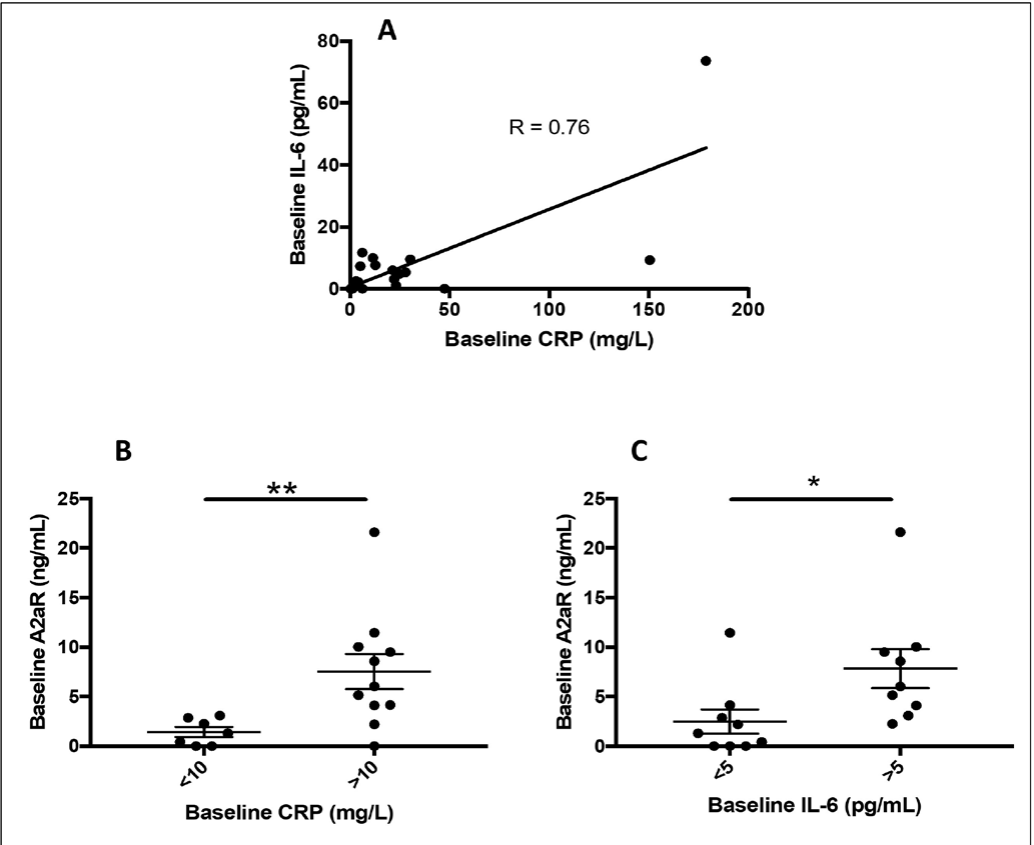
(A) Correlation of baseline IL-6 in the plasma with the blood CRP in n=18 patients in the East Carolina University cohort demonstrating a correlation coefficient (r) of 0.76. (B) Median levels of A2aR in the plasma were significantly higher for CRP-high versus CRP-low as well as in (C) patients with above and below median levels of IL-6 in the plasma. A2aR, adenosine 2a receptor; CRP, C-reactive protein; IL, interleukin.

In a prospective observational pilot study, we enrolled 18 patients with advanced NSCLC treated with ICIs to explore further the relationship between CRP, IL-6, and A2aR in plasma. In this cohort, two patients had stage IIIB disease, and 16 had stage IV disease at treatment initiation. Six patients were treated with Chemo-ICI and the remainder with single-agent ICI. The patients received a median of 4 ICI cycles (range: 1–8 cycles). In this cohort, the median baseline levels of CRP, IL-6, and A2aR were 16.9 mg/L, 5.1 pg/mL, and 3.6 ng/mL, respectively. Similar to the data of Keegan *et al*,^45^ a positive correlation was observed between baseline CRP levels and IL-6 plasma levels (R=0.76; p=0.003) (figure 3A). In addition, patients were stratified CRP-low (n=7) and CRP-high (N=11). As a result, CRP-high patients had a greater median baseline plasma A2aR level (6.0 ng/mL vs 1.30 ng/mL; p=0.006) (figure 3B). Likewise, nine patients had a baseline IL-6 level above the rounded median (≥5 pg/mL) that correlated with a higher median baseline level of A2aR (6.0 ng/mL vs 1.30 ng/mL; p=0.011) (figure 3C). In combination, these data reinforce the correlation of IL-6 and the adenosine pathway in advanced NSCLC and the use of high CRP as a surrogate for an immunosuppressive TIME (tumor immune microenvironment).

## Discussion

The impact of systemic inflammation on the prognosis of patients with advanced malignancies has been known for decades. However, attempts to elucidate therapeutically actionable drivers that can reverse the poor prognosis of patients with evidence of tumor-related inflammation have yet to materialize. Using a clinical and translational cohort, we demonstrate the role of inflammation-based markers such as CRP in patient stratification and a link between the IL-6 and adenosine pathways that may mediate resistance to ICI-based therapies.

In the observational study (cohort #1), the largest and most geographically heterogeneous to our knowledge, we demonstrate that elevated pretreatment CRP defines a subset of patients with NSCLC with poorer outcomes from ICI-based therapies. In the exploratory across ICI treatment strategies, we confirmed the significant stratification potential of baseline CRP levels in patients treated with chemotherapy-free ICI regimens, while the analysis fell short of statistical significance among patients treated with Chemo-ICI regimens, a finding that is most likely related to the limited sample size of the Chemo-ICI cohort, but which also allows us to speculate about a possible mitigating effect of the chemotherapy backbone against the negative prognostic role of the deranged systemic inflammation portrayed by high CRP levels. Others have shown that a decrease in on-treatment CRP correlates with improved PFS and responses to ICI in metastatic NSCLC.^45^ Similarly, a reduction in CRP during atezolizumab treatment for NSCLC was a strong surrogate for numerical PFS/OS benefit and better responses in both the OAK/POPLAR cohort and the B-F1RST trial.^46 47^ Thus, both baseline and on-treatment changes in CRP could serve as a biomarker for ICI-based approaches.

In addition to peripheral blood-based markers such as CRP, we sought to identify related biomarkers and therapeutic targets within the TIME. In a cohort of advanced NSCLC treated with pembrolizumab (cohort# 2; figure 2), we demonstrated that tumor IL-6 expression was associated with lower ORR and PFS. Paradoxically, using a deconvolution approach, we observed an opposing influence of IL-6 expression on T-cells, where tumors having responses to ICI had a trend for higher median IL-6 expression on T-cells (figure 2C). The pro-tumorigenic role of chronic IL-6 presence on the tumor compared with its role in promoting antitumor adaptive immunity via trans-signaling on immune cells that has been previously described,^48^ suggests a dual role of IL-6 in the TME.

Emerging data have provided mechanistic insights on the immune suppressive role of both elevated CRP and IL-6 on different facets of adaptive immunity.^49^ Elevated CRP was associated with an increased expression of negative checkpoints on CD8+T cells. Specifically, high CRP levels negatively affected calcium influx in T-cells, representing an important trigger of antigen major histocompatibility complex binding to T-cell receptors (TCRs), impairing effector T-cell function and antigen presentation. It is speculated that the downstream immunosuppressive effects in patients with elevated CRP could be due to IL-6 induction of STAT3, which can influence the expression of immunosuppressive cytokines such as transforming growth factor beta, suppress natural killer cell function and influence effector T-cell function, among other alterations within the TME.^50 51^ IL-6 also indirectly influences glucocorticoid levels in the body by transcriptional regulation of the peroxisome proliferator-activated receptor alpha, reprogramming host metabolism, thereby affecting the host antitumor immunity.^52^ Some of these findings were validated in hepatocellular carcinoma, where a high baseline IL-6 level was associated with worse ICI outcomes and resulted in attenuated T-cell immunity.^53^

Interestingly, anti-IL-6 receptor (anti-IL-6R) monoclonal antibodies can result in significant growth inhibition of certain NSCLC cell lines through activation of the NF-κB (nuclear factor kappa-light-chain-enhancer of activated B cell) pathway and inhibit lung cancer promotion in a *K-RAS* mutant mouse model by reprogramming the TME.^54 55^ Though multiple IL-6-mediated pathways may contribute to tumor growth and immune escape, our data demonstrate that elevated baseline tumor IL-6 expression could portend worse outcomes to ICIs in patients with NSCLC. Along with our data, these collective observations reinforce the premise for ongoing phase 2 trials combining tocilizumab, an anti-IL-6R targeted antibody with ICIs in melanoma (NCT03999749) and NSCLC (NCT04691817).

Our data showed enrichment of purine metabolism pathways within NSCLC tumors (cohort #2) that have higher expression of IL-6. This further strengthens the possibility of a relationship between the adenosine pathway and the IL-6 axis. It is plausible that the upregulation of purinergic signaling machinery within these tumors represents a compensatory mechanism to attenuate the pro-inflammatory processes triggered by hypoxia and IL-6 in the TME. Furthermore, induction of purinergic pathways leading to adenosine signaling has been seen to augment differentiation of M2, like immunosuppressive macrophages that are pro-tumorigenic.^56^ Thus, targeting this pathway could represent a potential approach to augment antitumor immunity in combination with ICIs.

To further evaluate the relationship of IL-6, CRP, and A2aR in the plasma, we conducted a prospective observational pilot study of 18 patients with NSCLC (cohort #3). We observed that high baseline plasma levels of CRP and IL-6 strongly correlated with elevated plasma levels of A2aR. Adenosine arises from the extracellular hydrolysis of adenosine triphosphate (ATP) by CD39 and CD73 on tumor cells. The adenosine signaling pathway promotes metastasis by enhancing cancer cell migration^57^ and, more importantly, suppressing antitumor immune cell functions through the direct inhibition of TCR activation and the increased transcription of immunosuppressive genes.^44^ Extracellular adenosine, often found in the TME, promotes immune suppression mainly through the A2aR expressed by immune cells within the peripheral blood mononuclear cells (PBMC) compartment.^58 59^ Some data suggest that soluble A2aR circulates as soluble tumor derived exosomes in the plasma.^60^ Similar to our approach, some other studies have employed this approach for A2aR biomarker assessment using ELISA to measure the receptor expression on cell surface.^59^ A2aR is an indirect surrogate for adenosine activity since measuring plasma adenosine can be challenging due to its short half-life and possible degradation.^61^ Currently, targeting the immunosuppressive signaling of adenosine through A2aR in the TIME is an area of active interest for cancer immunotherapy.^30^ Notably, a recent phase 1 clinical trial evaluating an A2aR antagonist for treatment-refractory renal cell carcinoma found that isolated human PBMCs stimulated with adenosine agonists produced increased amounts of IL-6.^43^ However, the exact mechanism remains unclear. We corroborated this analysis by demonstrating a positive correlation between tumorous IL-6 expression and a previously defined adenosine pathway signature in a TCGA cohort of lung adenocarcinoma (figure 2E). Others also noted that this specific adenosine signature has a strong positive correlation with a “myeloid inflammatory signature” in various solid tumors which was associated with poor responses to ICI combination with anti-angiogenic therapy.^62^

Due to the interlink between IL-6 and adenosine metabolism, our hypothesis-generating findings in cohorts #2 and #3 indicate a potential role of A2aR inhibition in conjunction with the anti-IL-6-R blockade in ICI refractory patients with a CRP-high phenotype. Furthermore, this strategy could overcome TIME factors associated with an impaired adaptive immune response in patients with NSCLC who do not respond optimally to first-line ICI-based therapies. Together, these findings support future studies elucidating the potential of IL-6 and the adenosine pathway as targets in NSCLC.

Overall, we propose that employing an integrated “multi-omics” approach to better understand the TIME in patients with NSCLC will help define rational treatment approaches with a high likelihood of success.^63 64^ In addition, such approaches will help improve outcomes by personalizing immunotherapeutic strategies with appropriate patient stratification. Examples of such approaches include the recently initiated Immuno-MATCH trial and the Keynote-495 trials, where whole-exome sequencing and gene-expression profiles are used to assign appropriate therapies to patients.^65 66^

Although these results are hypothesis-generating and could have important clinical implications, several limitations must be acknowledged. Beyond the retrospective design and relevant selection bias, the reduced sample size of subgroups, missing data, and inclusion of different tumor stages (stage III and IV) may have affected our analysis. In particular, missing baseline CRP values could have impacted our analysis of the included patients. Additionally, the high prevalence of data missingness for PD-L1 tumor expression and smoking status could have affected the distribution of these characteristics across subgroups. We also did not have CRP levels in cohort 2 to correlate these with IL-6 expression on the tumor. It is important to note that measuring A2aR in plasma is complex due to their membrane-bound nature. Although immunological approaches such as ELISA are useful, accurately studying these receptors in the plasma can be challenging and result in inaccuracies.

In conclusion, the potential influence of the immune suppressive effects of elevated CRP, IL-6, and the adenosine pathway on the antitumor efficacy of ICIs in NSCLC needs further evaluation and warrants prospective clinical trials targeting different facets of these pathways.

10.1136/jitc-2023-007310.supp1 Supplementary data

## Data Availability

Data are available upon reasonable request. De-identified data and data dictionary used may be made available at the request of investigators whose proposed use of the data has been approved by all investigators/authors.
