# Supplementary material for: Increased interleukin-6/C-reactive protein levels are associated with the upregulation of the adenosine pathway and serve as potential markers of therapeutic resistance to immune checkpoint inhibitor-based therapies in non-small cell lung cancer
Source: J Immunother Cancer. 2023 Oct 18;11(10):e007310. doi: 10.1136/jitc-2023-007310 (PMC10603340; doi:10.1136/jitc-2023-007310)
Supplement: Supplementary data [file jitc-2023-007310supp002.pdf]

Increased Interleukin-6 / C-reactive protein levels are associated with the upregulation of the adenosine pathway and serve as potential markers of therapeutic resistance to immune checkpoint inhibitor-based therapies in non-small cell lung cancer.

Naqash AR, et al.

Supplementary Table 1 Page 2

Supplementary Table 2 Page 3

Supplementary Figure 1 Page 4

Supplementary Figure 2 Page 5

Supplementary Table 1. Cohort #1 baseline patient characteristics across participating centres. ICL: Imperial College London; OYS: Oulu University Hospital; AQ: University Hospital of L'Aquila; ECU: East Carolina University.

Variable	**ICL** 57 **N (%)**	**OYS** 40 **N (%)**	**AQ** 41 **N (%)**	**ECU** 170 **N (%)**
**CRP** (mg/dl)				
Median (range)	25.1 (0.7-302.4)	17.5 (3-156)	21.5 (0.4-246.7)	20.9 (0.1-205)
**Age**				
≥ 65 years old	29 (50.9)	14 (35.0)	13 (31.7)	84 (49.4)
< 65 years old	28 (49.1)	26 (65.0)	28 (68.3)	86 (50.6)
**Gender:**				
Female	21 (36.8)	11 (27.5)	10 (24.4)	69 (40.6)
Male	36 (63.2)	29 (72.5)	31 (75.6)	101 (59.4)
**Histology**				
Non-Squamous	41 (71.9)	19 (47.5)	23 (56.1)	113 (66.5)
Squamous	16 (28.1)	21 (52.5)	18 (43.9)	57 (33.5)
**Cancer Stage**				
IIIb/IIIc	13 (22.8)	7 (17.5)	-	48 (28.2)
IV	44 (77.2)	33 (82.5)	41 (100)	122 (71.8)
**PD-L1%**				
0	6 (10.5)	-	9 (22.0)	32 (18.8)
1-49	16 (28.1)	6 (15.0)	10 (24.4)	13 (7.6)
≥50	25 (43.9)	15 (37.5)	8 (19.5)	29 (17.1)
Unknown	10 (17.5)	19 (47.6)	14 (34.1)	96 (56.5)
**Treatment line**				
First	10 (17.5)	13 (32.5)	6 (14.6)	48 (28.2)
Non-first	47 (82.5)	27 (67.5)	35 (85.4)	122 (71.8)
**Treatment Regimen**				
Chemo-ICI	1 (1.8)	3 (7.5)	4 (9.8)	40 (23.5)
ICI alone	56 (98.2)	37 (92.5)	37 (90.2)	130 (76.5)

Supplementary Table 2. Cohort #2 baseline patient demographics and biomarker data. The number in brackets next to each variable is the valid total for each category. The percentages calculated for each category are based on the valid total.

Variable	N (%)
**Age (59)**	
**<65**	39 (66.1)
**≥65**	20 (33.9)
**Race (59)**	
**Korea**	59 (100)
**Non-Korean**	0 (0)
**Gender (59)**	
**Male**	38 (64.4)
**Female**	21 (35.6)
**ECOG (59)**	
**≤1**	52 (88.1)
**>1**	7 (11.9)
**Histology (59)**	
**Non-Squamous**	59 (100)
**Squamous**	0 (0)
**CTx line number (59)**	
**0**	8 (13.6)
**1**	23 (39.0)
**2**	17 (28.8)
**≥3**	11 (18.6)
**Line of therapy (59)**	
**1**	8 (13.6)
**2**	23 (39.0)
**3**	17 (28.8)
**≥4**	11 (18.6)
**Subtypes (59)**	
ALK	4 (6.8)
EGFR	7 (11.9)
ERBB2	1 (1.7)
KRAS	6 (10.2)
MET	1 (1.7)
RET	1 (1.7)
ROS1	2 (3.4)
NA	37
**PFS (59)**	
**<50**	22 (37.3)
**≥50, <100**	11 (18.6)
**≥100**	26 (44.1)
**PD-L1 % (59)**	
**0**	12 (20.3)
**1-49**	15 (25.4)
**≥50**	21 (35.6)
**N/A**	11

Supplementary Figure 1. Study consort flow diagram showing patient distribution for clinical analyses.

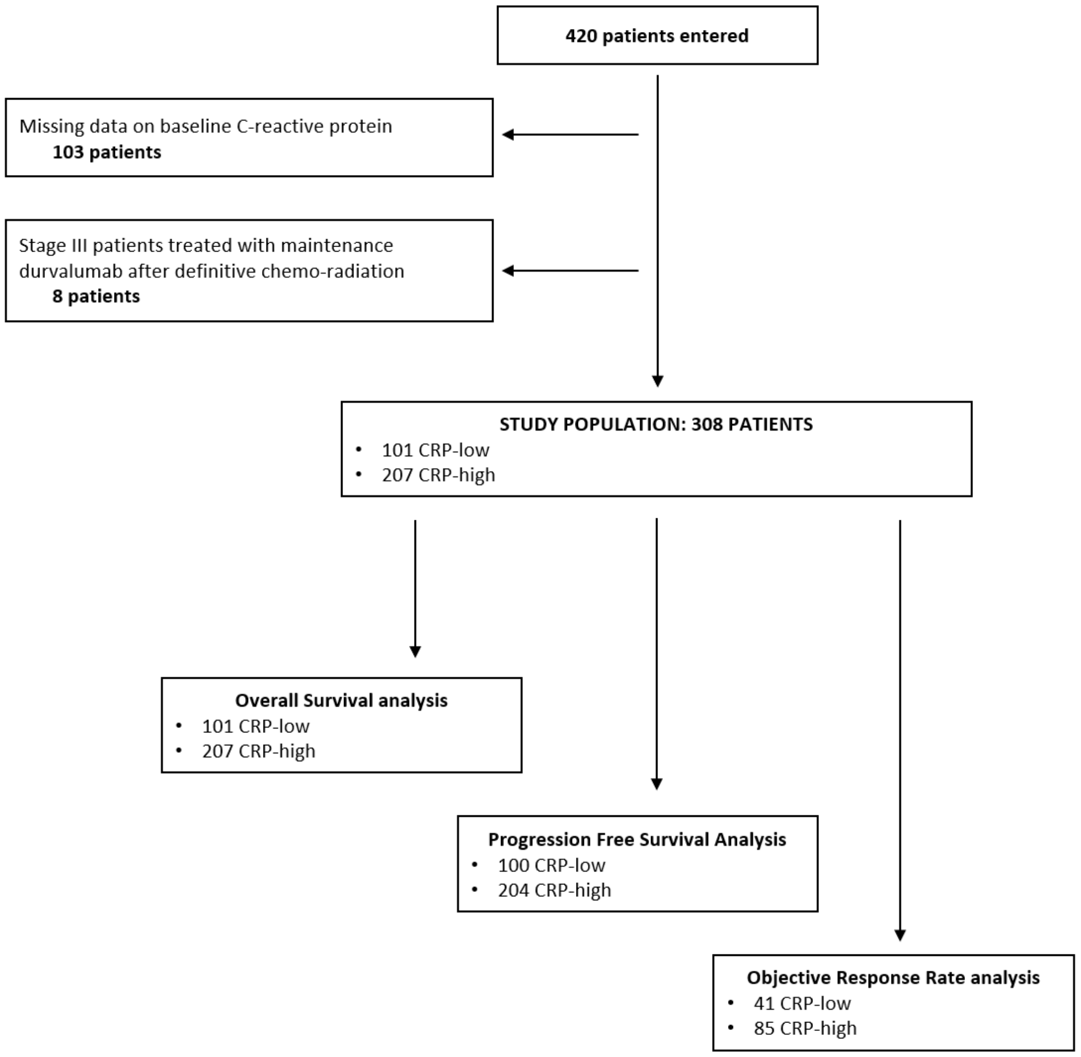

Supplementary Figure 2. Kaplan-Meier survival estimates among the ICI-alone and chemo-ICI cohorts. **A) Overall Survival (OS), ICI alone cohort.** The median OS for patients with CRP-high was 7.7 months (95%CI: 5.7-9.8; 126 events), while the median OS for CRP-low patients was 12.4 months (95%CI: 8.4-19.7; 49 events) (p=0.013) HR=1.51 (95%CI: 1.09-2.11). **B) Progression Free Survival (PFS), ICI alone cohort.** The median PFS for CRP-high patients was 3.6 months (95%CI: 2.9-4.2; 149 events), while for patients with CRP-low was 6.0 months (95%CI: 4.9-8.7; 62 events). (p=0.02) HR=1.42 (95%CI: 1.05-1.91). **C) OS, chemo-ICI cohort.** The median OS for patients with CRP-high was not reached (11 events) as for patients with CRP-low (3 events) (p=0.17). HR=2.38 (95%CI: 0.66-8.57). **D) PFS, chemo-ICI cohort.** The median PFS for CRP-high patients was 8.9 months (95%CI: 4.7- NA; 19 events), compared to 11.0 months (95%CI: 6.9-NA; 9 events) of CRP-low patients (p=0.51). HR = 1.29 (95%CI: 0.59-2.88).

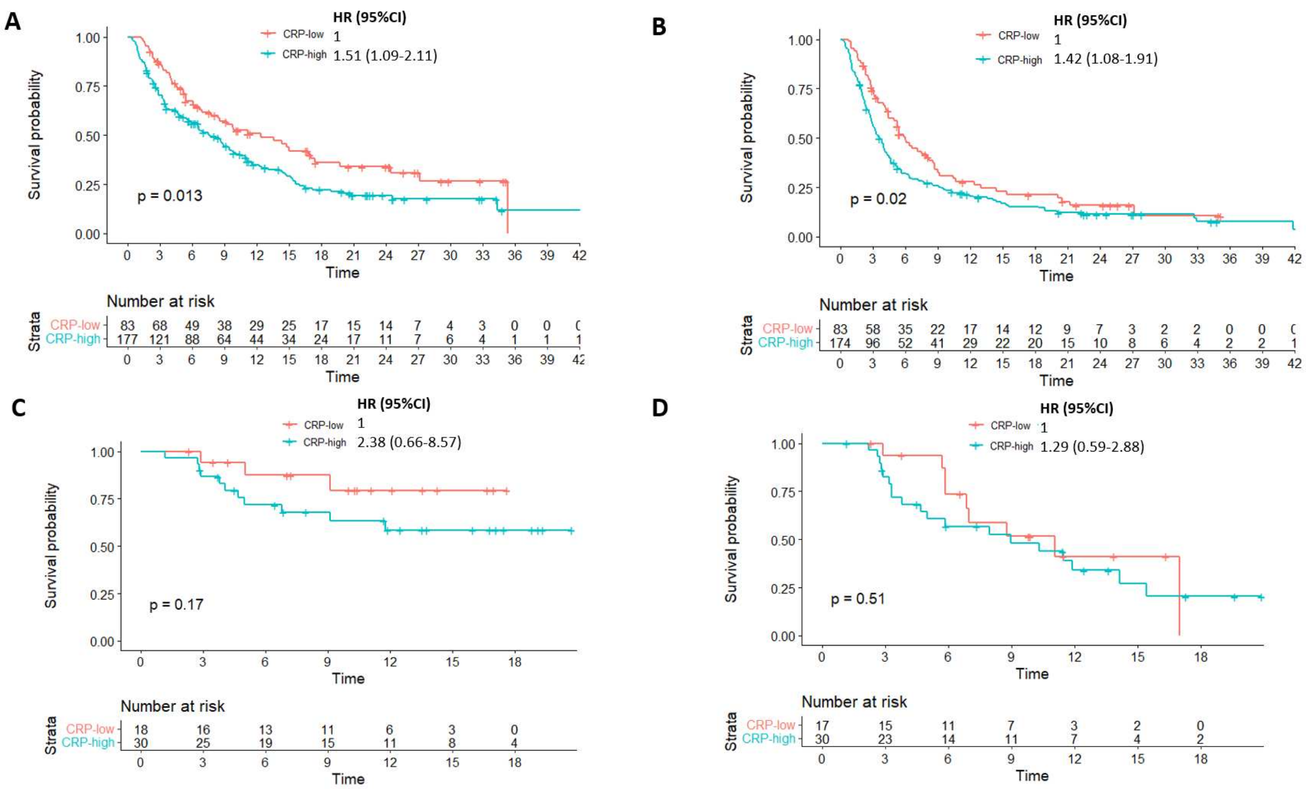

Naqash AR, *et al. J Immunother Cancer* 2023; 11:e007310. doi: 10.1136/jitc-2023-007310
