# Supplementary figures and images for: Increased interleukin-6/C-reactive protein levels are associated with the upregulation of the adenosine pathway and serve as potential markers of therapeutic resistance to immune checkpoint inhibitor-based therapies in non-small cell lung cancer
Source: J Immunother Cancer. 2023 Oct 18;11(10):e007310. doi: 10.1136/jitc-2023-007310 (PMC10603340; doi:10.1136/jitc-2023-007310)

Graphical abstract

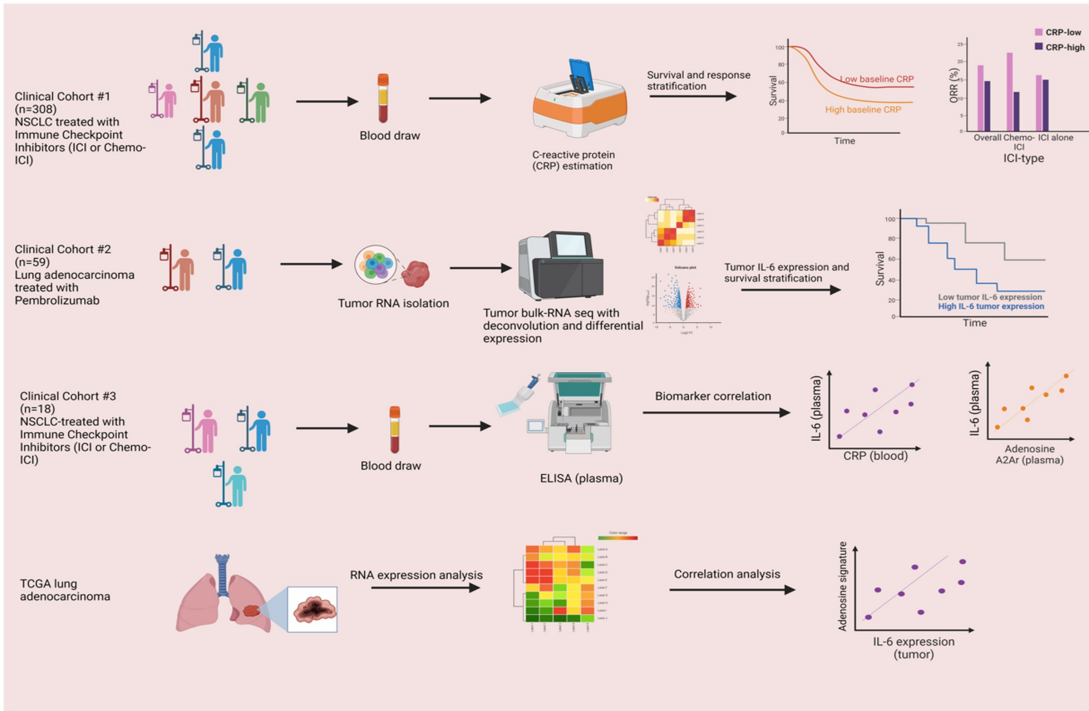

Created with BioRender.com

Supplement: Supplementary data [file jitc-2023-007310supp001.pdf]
